# FtsK, a DNA Motor Protein, Coordinates the Genome Segregation and Early Cell Division Processes in Deinococcus radiodurans

**DOI:** 10.1128/mbio.01742-22

**Published:** 2022-10-27

**Authors:** Shruti Mishra, Hari S. Misra, Swathi Kota

**Affiliations:** a Molecular Biology Division, Bhabha Atomic Research Centre, Mumbai, Maharashtra, India; b Life Sciences, Homi Bhabha National Institute, Mumbai, Maharashtra, India; University of Warwick; Massachusetts Institute of Technology

**Keywords:** *Deinococcus radiodurans*, bacterial cell division, FtsK, DNA translocation

## Abstract

Filament temperature-sensitive mutant K (FtsK)/SpoIIIE family proteins are DNA translocases known as the fastest DNA motor proteins that use ATP for their movement on DNA. Most of the studies in single chromosome-containing bacteria have established the role of FtsK in chromosome dimer resolution (CDR), connecting the bacterial chromosome segregation process with cell division. Only limited reports, however, are available on the interdependent regulation of genome segregation and cell division in multipartite genome harboring (MGH) bacteria. In this study, for the first time, we report the characterization of FtsK from the radioresistant MGH bacterium Deinococcus radiodurans R1 (drFtsK). drFtsK shows the activity characteristics of a typical FtsK/SpoIIIE/Tra family. It stimulates the site-specific recombination catalyzed by Escherichia coli tyrosine recombinases. drFtsK interacts with various cell division and genome segregation proteins of D. radiodurans. Microscopic examination of different domain deletion mutants of this protein reveals alterations in cellular membrane architecture and nucleoid morphology. *In vivo* localization studies of drFtsK-RFP show that it forms multiple foci on nucleoid as well as on the membrane with maximum density on the septum. drFtsK coordinates its movement with nucleoid separation. The alignment of its foci shifts from old to new septum indicating its cellular dynamics with the FtsZ ring during the cell division process. Nearly, similar positional dynamicity of FtsK was observed in cells recovering from gamma radiation exposure. These results suggest that FtsK forms a part of chromosome segregation, cell envelope, and cell division machinery in D. radiodurans.

## INTRODUCTION

Bacteria orchestrate their cell division in time and space by the coordinated action of proteins involved in cell division, named the divisome, and genome maintenance, named the segrosome. The divisome assemblies contain more than 20 proteins in a variety of bacteria like Escherichia coli, Neisseria gonorrhoeae, Pseudomonas aeruginosa, etc. (1–3), which ensures an accurate cell constriction, septal peptidoglycan (PG) synthesis, and, finally, cell separation (4). One of these proteins, filament temperature-sensitive mutant K (FtsK), was first documented in a temperature sensitive mutant of E. coli TOE44 that was impaired in cell division. It was observed that, at 42°C, its mutant forms highly indented filaments suggesting blockage at a very late stage of division, and was named filament temperature-sensitive mutant K TOE44 (AB2497 ftsK44) (5). Later, cellular localization studies revealed that FtsK foci form on the septum of E. coli (6), and this localization of FtsK, along with FtsA, recruits other cell division proteins like FtsL, FtsQ, and FtsI, which are required for the stabilization of divisome formed at the mid-cell position (7). FtsK was characterized as a multifunctional DNA translocase belonging to the additional strand conserved E (ASCE) family P-loop NTPases (8), involved, not only in the stabilization of divisional septa and DNA translocation, but also in membrane synthesis and cell envelope remodeling by interacting with various proteins like septal peptidoglycan binding protein rare lipoprotein A (RlpA) and proteins involved in peptidoglycan synthesis like FtsI (9, 10). The protein is divided into 3 domains- the N-terminal domain of around 200 amino acids (NTD), the linker region (FtsKL), and the 500 amino acids C-terminal domain (CTD) (11, 12). In E. coli, the NTD is responsible for the attachment to the cell membrane by transmembrane (TM) segments and the interaction with other cell division and cell envelope proteins (13). The FtsKL is not conserved and varies in length and composition between species (14, 15). The CTD is highly conserved and comprises 3 separate subdomains called α, β, and γ (16). This region is known for the motor functions of FtsK and helps in DNA translocation. The γ domain (FtsKγ) is attached to the β-domain via a flexible linker and recognizes 8-bp sequences called FtsK Orienting Polar Sequence (KOPS)(GGGNAGGG) in E. coli and SpoIIIE recognition sequence (SRS) (GAGAAGGG) in Bacillus subtilis. KOPS/SRS sequences are asymmetrically distributed on the chromosome arms, and direct the translocation of FtsK/SpoIIIE/Tra family proteins toward the deletion-induced filamentation (*dif*) site in the “*ter”* region (17, 18). In this region, 2 ‘*dif*’ sites are brought closer by FtsK which thereby recruits and activates tyrosine recombinases XerCD to form the synaptic complex XerCD-*dif* (19). This XerCD-*dif* complex is involved in site-specific recombination (SSR) to resolve the chromosome dimers (chromosome dimer resolution- CDR) (20, 21). In this way, FtsK helps in sorting duplicated chromosome copies at the site of cell division (22).

Deinococcus radiodurans R1 is a coccus-shaped Gram-positive bacterium that shows extreme resistance to multiple abiotic stresses like gamma radiation, desiccation, and oxidative stress (23, 24). These phenotypes are attributed to its highly efficient DNA double-strand break repair and a strong anti-oxidant mechanism (25, 26). This bacterium exists in tetrads and consists of a multipartite genome system (MGS) with 2 chromosomes (Chr I, Chr II) and 2 plasmids (Mega plasmid [MP] and small plasmid [SP]). It has a polyploid multipartite genome, which is highly catenated and intertwined, making the nucleoid tightly arranged in the form of a very distinct doughnut-shaped toroidal structure (27). How this genome arrangement is maintained and segregated during cell division is still unknown. The D. radiodurans genome encodes putative FtsK/SpoIIIE family proteins (28). In what way FtsK might act to help segregate multiple, complex chromosomes is an outstanding question of great interest. Here, we report the characterization of FtsK of D. radiodurans (drFtsK) and its potential role in genome separation, septum formation, and cell division. We demonstrated that the purified drFtsK showed concentration-dependent ATPase activity, sequence-specific interaction with KOPS DNA of E. coli, and could stimulate SSR by E. coli XerCD *in vitro*. Protein-protein interaction studies indicated that drFtsK interacts with various genome segregation and cell division proteins. Further, different domain deletion mutants of dr*ftsK* exhibited a significant change in the growth rate under normal conditions as well as after irradiation during the post-irradiation recovery (PIR) period. Morphological studies indicated that the deletion of different domains of drFtsK affected the nucleoid arrangement and cellular architecture in this bacterium resulting in some abnormal phenotypes. The localization studies of FtsK-RFP expressed under native promoter showed the appearance of dispersed fluorescent foci on the nucleoid, septal, and peripheral membranes of the cells and a coordinated movement with FtsZ during cell growth. Together, these results suggest that drFtsK helps in cross-talks between cellular and molecular events like genome segregation, cell envelope remodeling, and septum formation in D. radiodurans.

## RESULTS

### The D. radiodurans genome encodes an FtsK homologue.

**FIG 1:**
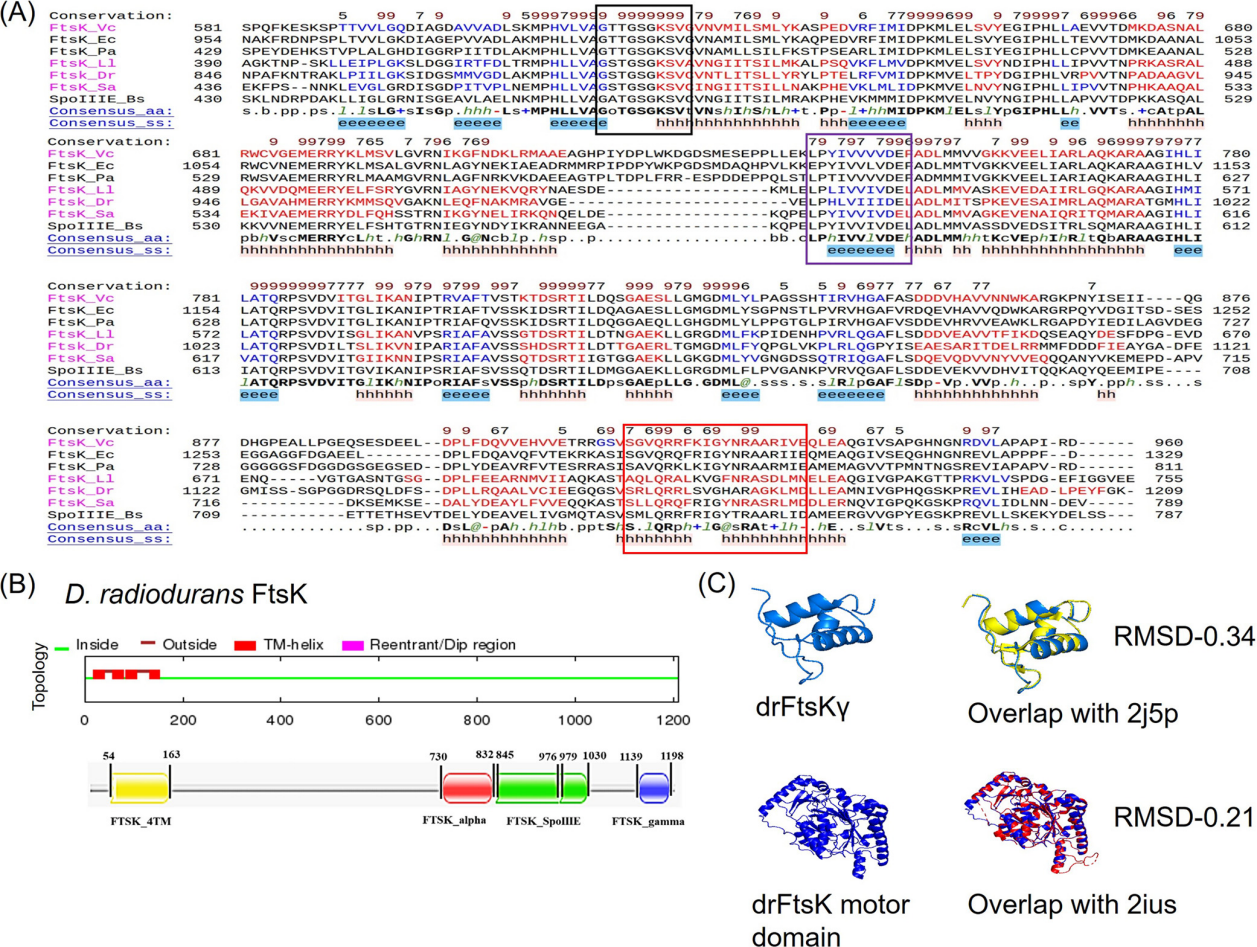
Deinococcal FtsK protein sequence alignment and modeling. (A) Multiple sequence alignment (MSA) of drFtsK with known FtsK/SpoIIIE family proteins. The amino acid sequences of FtsK from Deinococcus radiodurans (FtsK_Dr), SpoIIIE from Bacillus subtilis (SpoIIIE_Bs), FtsK of Escherichia coli (FtsK_Ec), Pseudomonas aeruginosa (FtsK_Pa), Lactococcus lactis (FtsK_Ll), Vibrio cholerae (FtsK_Vc), and Staphylococcus aureus (FtsK_Sa) are collected from NCBI and the homology between sequences is checked by PROMALS3D webserver. MSA of the C-terminal region is depicted here. Boundaries of the conserved motifs in the C-terminal are marked as a black box for the walker A domain (ATP binding P-loop motif), a purple box for the Walker B motif, and a red box for the DNA binding motif. Predicted secondary structures are displayed below the sequences. (B) Different domains present in drFtsK are represented as FTSK_4TM (FtsK_N-terminal region), FtsK_alpha, FtsL_SpoIIIE (αβ motor pump) and FtsK_gamma domain(drFtsKγ). (C) Modeled structures of drFtsKγ and motor domain were aligned with the template structures of E. coli FtsK gamma domain (2j5p) and E. coli FtsK motor domain (2ius), respectively.

Upon BLAST search, we found that the putative deinococcal FtsK (drFtsK) was wrongly annotated in the genome of D. radiodurans. Indeed, the coding sequence of putative drFtsK spans upon 2 Open Reading Frames (ORF) i.e., Dr_0400 and Dr_0401 in chromosome I. Dr_0401 contains the N-terminal region (229 amino acids) and Dr_0400 contains the remaining region (980 aa) (28). Recently, this has been corrected and the entire coding sequence of FtsK is annotated with a locus tag- E5E91_ RS02025 (29). Multiple sequence alignment using the PROMALS3D tool showed that drFtsK has ~25-30% identity with FtsK/SpoIIIE members of other species (E. coli [FtsK_Ec], V. cholerae [FtsK_Vc], P. aeruginosa [FtsK_Pa], L. lactis [FtsK_Ll], S. aureus [FtsK_Sa], B. subtilis [SpoIIIE_Bs]). The overall protein homology is less because the N-terminal and linker region is quite variable, but the C-terminal region is highly conserved (Fig. 1A and data at https://barc.gov.in/publications/mbio/dna_mp/mBio01742-22R1.pdf). Consensus sequences for ATP binding P-loop motif (Walker A motif), Walker B motif, and winged helix-turn-helix (wHTH) DNA binding region were compared with the drFtsK sequence. The results showed that the C-terminal domain of drFtsK contains a consensus ATP binding motif and DNA binding motif indicating it is an FtsK homologue (Fig. 1A). Further, the phylogenetic analysis revealed that drFtsK forms a separate clade (see the data at https://barc.gov.in/publications/mbio/dna_mp/mBio01742-22R1.pdf). Domain prediction exhibited that drFtsK contains the canonical FtsK domains- FTSK_4TM (FtsKN), FtsK_alpha, FtsK_SpoIIIE (αβ motor pump) and FtsK_gamma domain (drFtsKγ) (Fig. 1B). Modeled structures of the drFtsK gamma and motor domains were aligned with the template structures of gamma (2j5p) and motor (2ius) domains in E. coli FtsK, respectively. The corresponding aligned models showed RMSD values of 0.21 (TM score 0.938) and 0.34 (TM score 0.962), respectively, indicating that these structures are highly similar (Fig. 1C). This information led us to hypothesize that drFtsK may perform similar functions as that of characterized FtsK/SpoIIIE proteins from other bacteria, provided that this bacterium has the functional homologs of tyrosine recombinases.

We also searched for putative tyrosine recombinases taking E. coli XerCD-specific domains for analysis. Six putative ORFs, in chromosome I- E5E91_RS02620 (old locus tag- Dr_0513); in chromosome II- E5E91_RS13705 (old locus tag- Dr_A0075), E5E91_RS14115 (old locus tag- Dr_A0155) and E5E91_RS14250 (old locus tag- Dr_A0182); in megaplasmid- E5E91_RS15640 (old locus tag- Dr_B0104) and in small plasmid- E5E91_RS15930 (old locus tag- Dr_C0018) were identified (see the data at https://barc.gov.in/publications/mbio/dna_mp/mBio01742-22R1.pdf). Which combination of these ORFs would function as tyrosine recombinases and is significant in genome segregation in this bacterium is worth understanding and will be addressed separately.

### drFtsK shows ATPase activity.

**FIG 2:**
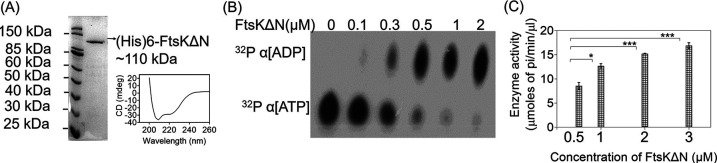
The ATPase activity of drFtsK. (A) Polyhistidine tagged fusion protein of N-terminal truncated protein (FtsKΔN) was purified and the purified protein was checked for proper refolding by circular dichroism as described in the methods. (B) ATPase activity of recombinant purified FtsKΔN protein was checked using radiolabeled ATP [^32^P]-αATP. Different concentrations of the protein were incubated with radiolabeled nucleotide and reaction products were separated on PEI-Cellulose F^+^ TLC. The autoradiogram shows the hydrolysis of ^32^P-α[ATP] to ^32^P-α[ADP]. (C) Quantitative analysis of ATPase activity was done by colorimetric malachite green reagent using increasing protein concentration. Data shown here are the mean+-SD (*n* = 3) plotted in GraphPad Prizm6. Statistical significance was obtained by Student's *t* test. The *P*-values attained at 95% confidence intervals are depicted as (***) for <0.001 and (*) for <0.05.

The N-terminal truncated drFtsK contains all functional domains like ATP and DNA interacting motifs, and gamma domain and lacks transmembrane segments that would be required for localization and *in vivo* function. So, N-terminal truncated drFtsK (drFtsKΔN) could be used for biochemical characterization *in vitro*. Therefore, we checked the ATPase activity and DNA binding activity of purified drFtsKΔN. Circular dichroism (CD) analysis showed that purified drFtsKΔN does contain folded protein and the protein has majorly α-helical conformation (Fig. 2A), which was supported by *in-silico* secondary structure prediction by PROMALS3D (see the data at https://barc.gov.in/publications/mbio/dna_mp/mBio01742-22R1.pdf). Further, the oligomeric status of the protein was checked by dynamic light scattering. Results showed that the protein exists in hexameric form even in the absence of DNA (see the data at https://barc.gov.in/publications/mbio/dna_mp/mBio01742-22R1.pdf). This indicates that drFtsK binds to DNA in a pre-formed hexameric form as shown previously for SpoIIIE in B. subtilis (30). The ATPase activity of purified drFtsKΔN was determined using [^32^P]-αATP hydrolysis (Fig. 2B) as well as a colorimetric malachite green ATPase activity assay (Fig. 2C). Results showed that drFtsKΔN is an ATPase that could convert ATP to ADP and inorganic phosphate. An increase in the release of inorganic phosphate was seen with the increase in protein concentration. However, the specific activity of the enzyme remained unaltered. Further, the presence of E. coli KOPS containing DNA could marginally stimulate the ATPase activity of purified drFtsKΔN (see the data at https://barc.gov.in/publications/mbio/dna_mp/mBio01742-22R1.pdf).

### drFtsK binds to E. coli KOPS and activates site-specific recombination catalyzed by tyrosine recombinases -XerCD.

**FIG 3:**
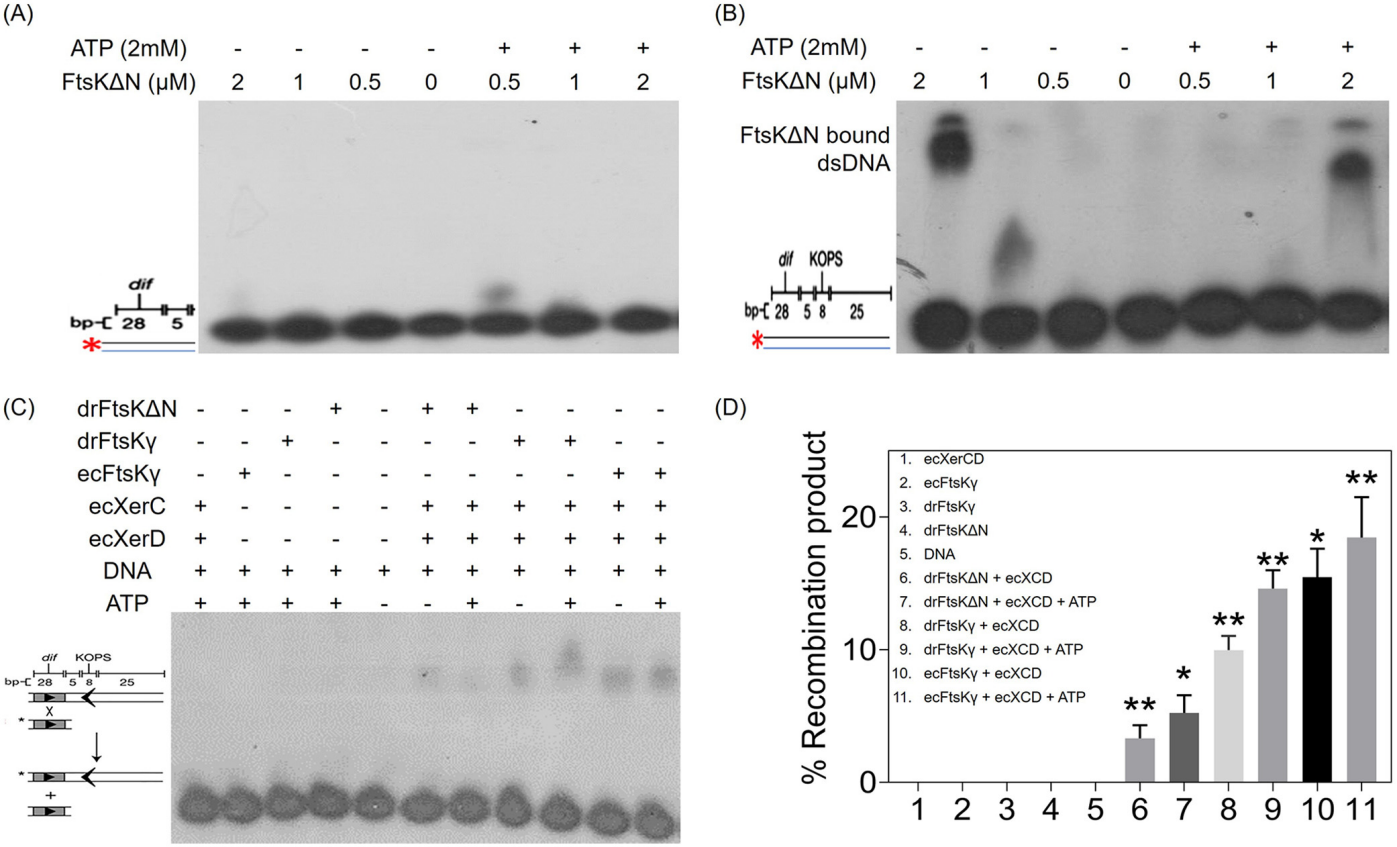
DNA binding activity and activation of E. coli tyrosine recombinases by deinococcal FtsKΔN protein. Purified recombinant FtsKΔN protein was checked for DNA binding activity with [*γ* -32P] ATP labeled dsDNA containing E. coli *dif*- 40 bp (A), E. coli *dif* and KOPS- 72 bp (B) in the presence/absence of ATP. Autoradiograms show EMSA gels where no interaction of drFtsKΔN is seen with only E. coli *dif* sequence but interaction with E. coli *dif* and KOPS is seen. A schematic representation of the recombination reaction substrate (short radiolabeled *dif* containing dsDNA sequence) and product (long *dif* + KOPS containing dsDNA sequence) as described earlier is given (31) (C). Autoradiogram showing site-specific recombination reaction by E. coli tyrosine recombinases XerC and XerD (ecXerCD). Recombination products were obtained in those reactions where FtsK (drFtsKΔN/drFtsKγ/ecFtsKγ) was present with EcXerCD. Band intensities obtained by densitometric analysis of the autoradiogram were used to calculate % recombination product in each reaction (D). Data shown here are the mean+-SD (*n* = 3) and statistical significance was found using the Student's *t* test. The *P*-values attained at 95% confidence intervals are depicted as (*) for <0.05 and (**) for 0.05-0.001.

It has been known that the FtsK protein of E. coli (ecFtsK) binds to KOPS and the orientation of these motifs on the chromosome is highly skewed so that they direct the FtsK translocase toward the terminal replichore region for decatenation of the duplicated circular chromosome. Several key residues implicated in KOPS recognition have been identified in the ecFtsK gamma domain especially S766, Q769, and N777 (31). We found that these residues are not identical across FtsK/SpoIIIE family proteins, which could lead to differences in the FtsK recognition sites in different organisms. Therefore, we analyzed the distribution of a few KOPS octamers in the deinococcal genome using DistAMo online tool. The results indicated that there is a high frequency of GGGNAGGG motif family and E. coli KOPS-GGGCAGGG on deinococcal chromosomes I with high density existing near the ‘ter’ region. Compared to chromosome I, chromosome II has less density (see the data at https://barc.gov.in/publications/mbio/dna_mp/mBio01742-22R1.pdf). Thus, over- or under-representation of these motifs suggests that the GGGCAGGG motif might function as KOPS in D. radiodurans. Compared to E. coli, however, the overall frequency of the GGGCAGGG motif is much less in the D. radiodurans genome. However, we monitored the DNA binding activity of drFtsKΔN by EMSA using radiolabeled dsDNA sequences containing E. coli *dif* + KOPS. The protein showed a sequence-specific binding with dsDNA containing the GGGCAGGG motif (Fig. 3B). The size of the nucleoprotein complex increased gradually (reflected as slower mobility) with the rise in protein concentration suggesting more FtsK molecules are binding to KOPS. Earlier, it was shown that in general, 3 FtsKγ domains bind to 8 bp KOPS DNA (31). The presence of ATP did not affect drFtsKΔN binding with KOPS sequences. This shift is not observed in the case of dsDNA of relatively shorter length, containing only the E. coli *dif* site without KOPS (Fig. 3A), suggesting that drFtsK interaction is specific to GGGCAGGG-KOPS that could act as the loading site for drFtsK on the genome in D. radiodurans.

As drFtsKΔN showed binding with KOPS, we tested the ability of drFtsKΔN and drFtsKγ to activate XerCD site-specific recombination (SSR). For this, the well-characterized E. coli recombination system components were utilized and an *in vitro* recombination assay was carried out using purified ecXerC (~34kDa) and ecXerD (~34kDa) proteins (see the data at https://barc.gov.in/publications/mbio/dna_mp/mBio01742-22R1.pdf) as described previously (31) and represented in Fig. 3C. Results showed that drFtsKΔN and drFtsKγ can stimulate ecXerCD mediated recombination between 2 dsDNA substrates- one containing only *dif* sites and the other containing *dif +*KOPS (Fig. 3C). Both drFtsKΔN and drFtsKγ showed a nearly similar pattern of recombination products in the presence and absence of ATP. The recombination at *dif* sites in the presence of drFtsKΔN and drFtsKγ proteins could be possible only when the γ domain in these proteins interacts with the ecXerCD and stimulates recombination activity. Upon quantification of the percent recombination product, it was noticed that the recombination reaction has occurred at low efficiency in the presence of drFtsKΔN. With drFtsKγ, it was found to be nearly similar to that observed with ecFtsKγ (recombinant protein containing E. coli FtsK gamma domain) which was used as a positive control (Fig. 3D). These results together suggested that drFtsKΔN and drFtsKγ proteins are functional at least *in vitro*, and exhibit ATPase, E. coli KOPS binding, and stimulate tyrosine recombination by E. coli XerCD at E. coli *dif* sites.

### FtsK deletion affects growth rate and morphology in D. radiodurans.

**FIG 4:**
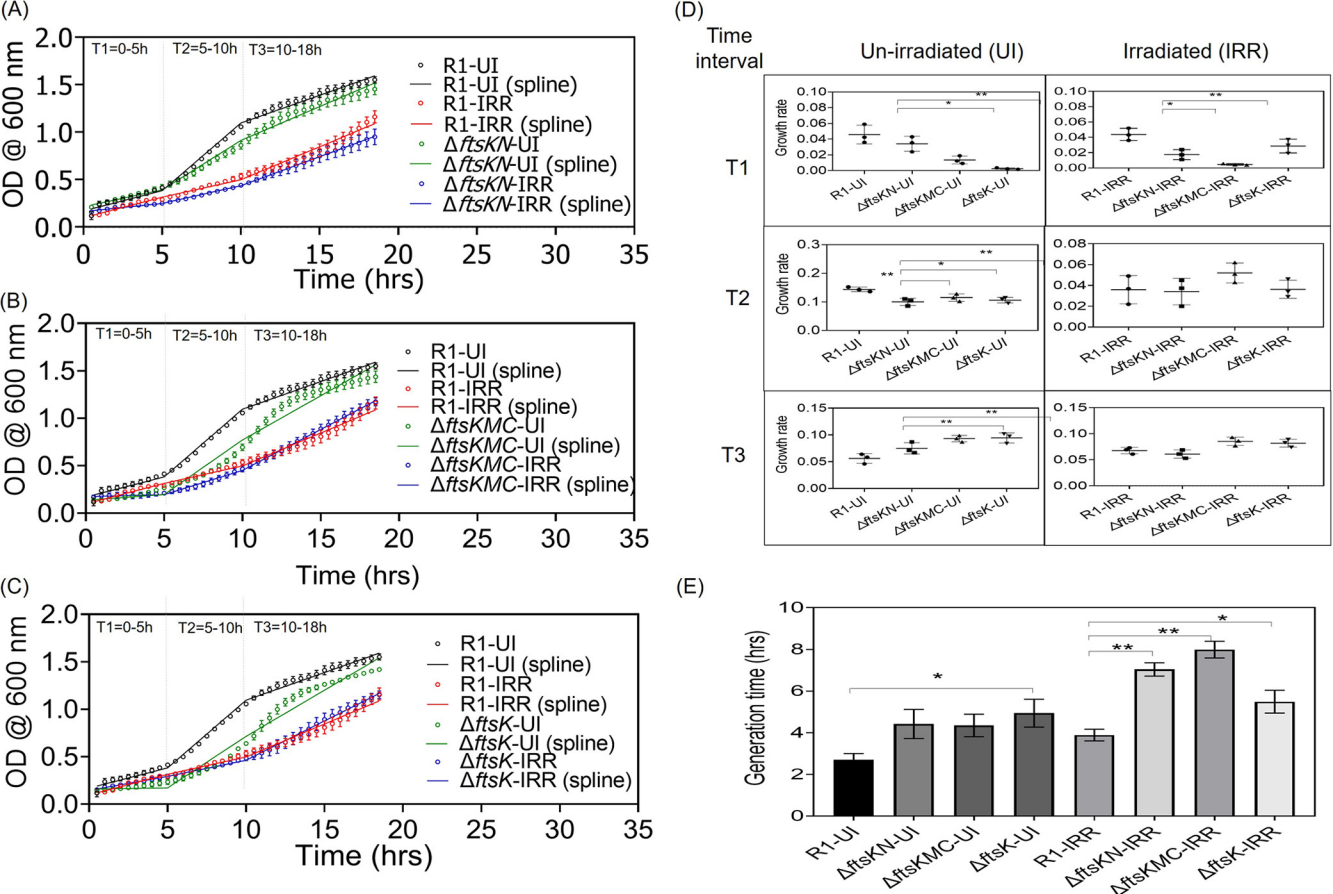
Effect of deinococcal FtsK deletion on the growth of D. radiodurans. Cell survival studies of different deletion mutants of *ftsk-ΔftsKN* (A), *ΔftsKMC* (B), and *ΔftsK* (C) were monitored under un-irradiated conditions (normal) and after gamma irradiation treatment (6kGy). The growth data (circles) were fitted by a linear spline regression model (lines). The dashed vertical lines show the knots dividing the spline modeled growth curve into three intervals- 0 to 5 h (T1), 5 to 10 h (T2), and 10 to 18 h (T3). The difference in the growth rates of wild-type (R1) and different domain deletion mutants under normal and post-irradiation recovery conditions at each time interval was calculated by estimating the slopes of each line segment from the spline regression (D). The generation time (doubling time) of wildtype and *ftsk* mutants under normal and irradiated conditions was calculated as described in the methodology and plotted (E). Data shown here are the mean+-SD (*n* = 9) and the statistical significance of the differences was found using the Student's *t* test. The *P*-values attained at 95% confidence intervals are depicted as (*) for <0.05 and (**) for 0.05-0.001.

To understand the *in vivo* roles of drFtsK, we created chromosomal deletions of the different regions in the coding sequences of this protein, and the effects of these deletions on growth and morphology were examined. We analyzed the growth curve data where the entire growth period was divided with 2 knots giving the time intervals- T1 (0 to 5 h), T2 (5 to 10 h), T3 (10 to 18 h) corresponding to lag, log, and stationary phases of growth, respectively, as defined in the methods. The D. radiodurans cells containing the deletion of full-length FtsK (Δ*ftsK*), middle and C-terminal domain (*ΔftsKMC*), and N-terminal domain (*ΔftsKN*) (see the data at https://barc.gov.in/publications/mbio/dna_mp/mBio01742-22R1.pdf) showed a differential effect on growth under un-irradiated (UI) conditions (Fig. 4). For instance, under UI, Δ*ftsKN* showed a significant change in the growth compared to wild type (WT) during T2 (Fig. 4A and 4D). On the other hand, *ΔftsKMC* and *ΔftsK* mutants showed extended lag period (Fig. 4B, C, and 4D) but showed faster growth during T3 (Fig. 4B, C, and 4D). This can be understood as the mutants might have not reached the stationary phase because of the slow growth rate and the extended lag phase during T1 and T2 (Fig. 4B and 4C). So, we compared the growth rate of mutants in T3 to that of the WT in T2. The growth rate of the mutants in the T3 (0.075 ± 0.01-Δ*ftsKN*, 0.093 ± 0.006-*ΔftsKMC*, 0.094 ± 0.009*-ΔftsK*) was still lower than that of WT in T2 (0.142 ± 0.008). During the post-irradiation recovery period (IRR), the effect of drFtsK domains deletion on the growth rate is different from unirradiated conditions. The growth of *ΔftsKMC* and *ΔftsK* mutants is significantly slower than the WT (R1) and Δ*ftsKN* during T1. The decreased growth rate was observed particularly during the lag phase under the post-irradiation recovery phase and normal growth conditions, where cellular processes like DNA damage repair, genome duplication, segregation, and cell division are actively occurring. This would have increased the generation time of the mutants (Fig. 4E). The slow growth rate in mutants indicated that FtsK may play some critical role in the normal cellular processes like genome segregation and cell division in this bacterium.

**FIG 5:**
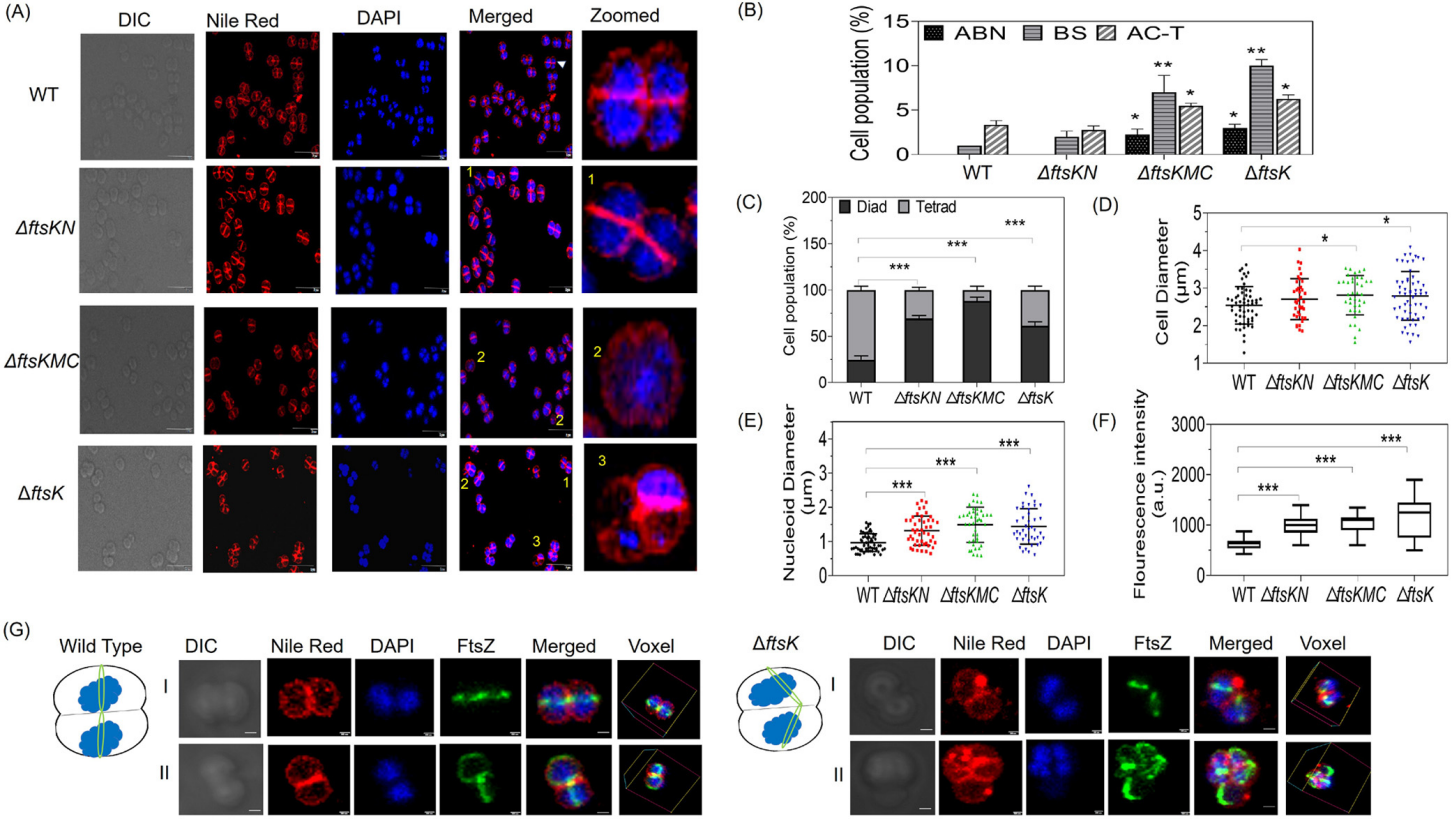
Effect of different domain deletion of *drftsK* on the phenotype of D. radiodurans cells. Cells at 14 h post subculturing were taken and fluorescence microscopic images show the DIC, TRITC (for Nile Red), DAPI, and merged channels of the wild type (WT), and different mutants of *ftsk*-*ΔftsKMC*, *ΔftsKN*, *ΔftsK* cells (scale bar-5μm). A significant population of cells in *ΔftsKMC*, *ΔftsK*, and *ΔftsKN* mutants showed a change in tetrad cell morphology, nucleoid arrangement, and septal membrane. Atypical phenotypes obtained are represented as; 1. Bent septum (BS), 2. Abnormal tetrad arrangements (ABN), and 3. Anucleated cells within the tetrad (AC-T) (zoomed cell scale bar- 500 nm) (A). Statistical analysis of these morphologies obtained in *ftsK* mutants when compared to WT cells is done in 200–300 cells and plotted (B). Other phenotypic changes like percent of diads and tetrads in the cell populations (C), cell diameter (D), nucleoid diameter (E), and nucleoid DAPI fluorescence intensity (F), and were calculated and plotted. The *P*-values attained at 95% confidence intervals are depicted as (*) for <0.05, (**) for 0.05-0.001, (***) for <0.001. Microscopic images of the cell division septal ring formation by drFtsZ-GFP in WT and *ΔftsK* mutant cells are depicted along with pictorial representation (scale bar- 500 nm) (G). The images shown here are representative pictures of the experiments conducted at least three times.

Further, we compared the cell morphology of *ftsK* deletion mutants with WT under non-irradiated (normal) conditions at 14 h post subculturing. The FtsK mutants, *ΔftsKN*, *ΔftsKMC*, and *ΔftsK* showed statistically significant changes in their cellular parameters like percent of tetrads and diads in a population, cell diameter, nucleoid compaction (as determined by nucleoid diameter), and nucleoid intensity (as determined by measuring DAPI fluorescence intensity). These phenotypes were quantified and compared to the wildtype (Fig. 5C, D, E, and 5F). The mutants showed a significantly higher number of diads than tetrads compared to the WT. Also, the mutant cells showed bigger cell and nucleoid diameter, and higher DAPI fluorescence intensity compared to the WT cells (Fig. 5A and 5B). Besides these, around ~10% cell population of the mutants showed atypical cellular morphologies like abnormal tetrad arrangements (ABN), bent septum (BS), and anucleated cells within the tetrad (AC-T). An increase in the nuclear parameters like diameter and DAPI intensity suggests a putative role of drFtsK in genome segregation and chromosome organization, while changes in the cellular parameters in mutants, like cell diameter, indicate growth/cell division impairment. To understand these phenotypes further, we expressed FtsZ, a major cell division protein, as FtsZ-GFP (pVHZGFP) in *ΔftsK* mutant, and cells were monitored for changes, if any, in FtsZ functions. We observed that a significant population (~10%) in *ftsK* deletion mutant got affected in the positioning of the FtsZ ring in D. radiodurans, as seen by FtsZ-GFP ring misplacement (Fig. 5G). Earlier, it was shown that deletion of the C-terminal region of DivIVA protein in D. radiodurans generates the bent septum (32). So, the presence of bent septum in *ΔftsK* mutant may be attributed to its probable role in the positioning of the pole determining protein and divisome components (like FtsZ) in this bacterium. In addition to bent septum and defective FtsZ ring formation, we also found that the FtsK mutants formed membrane bulges and showed changes in membrane staining properties at exponential phase time points of 6 and 9 h of subculturing. As shown at the 6 h time point, all the mutants showed membrane bulging in a significant number of cells. Notably, the *ΔftsKN* mutant cell was able to mostly recover from this phenotype at 9 h, but the other 2 mutants could not recover, indicating the role of drFtsK in membrane biology in D. radiodurans (see the data at https://barc.gov.in/publications/mbio/dna_mp/mBio01742-22R1.pdf). These phenomena also coincide with the extended lag period and increased growth rate during initial growth periods, further suggesting the drFtsK role in bacterial cell cycle processes. Further, the survival of cells upon *ftsK* deletion, even after significant phenotypic changes, might argue its functional redundancy in this bacterium.

### FtsK localizes on nucleoid and septum in D. radiodurans.

**FIG 6:**
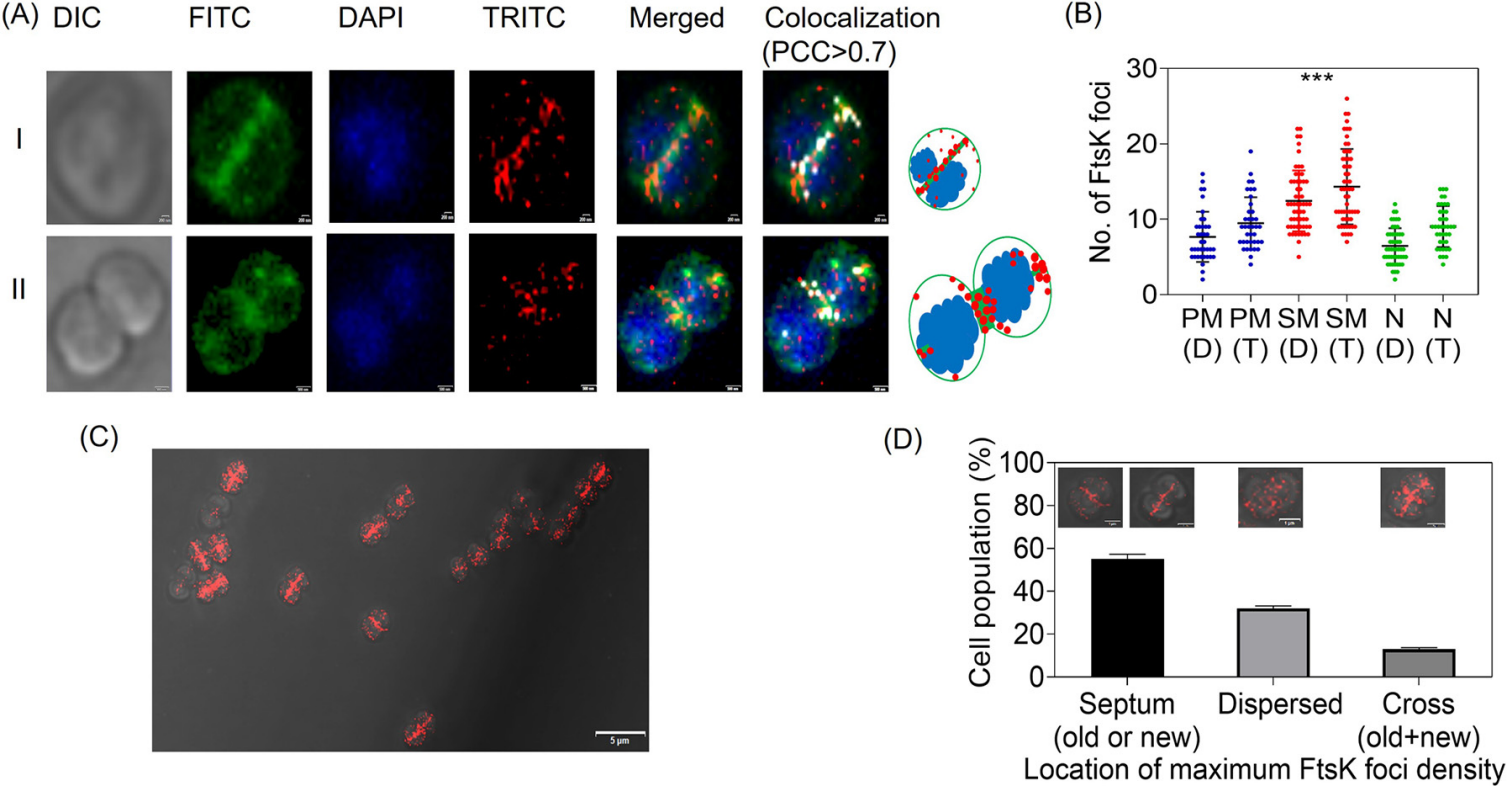
Cellular localization of FtsK-RFP expressing under native promoter in D. radiodurans *cells*. Expression of FtsK-RFP foci (TRITC channel- red) in the cells is seen after nucleoid staining by DAPI (blue) and membrane staining by Vancomycin-BioDIPY (FITC channel- green). FtsK-RFP shows association on the nucleoid as well as on the membrane, both septal and peripheral membrane. Data represented here shows microscopic images of two independent cells along with pictorial images (scale bar- 200 nm and 500 nm for I and II, respectively) (A). White foci show the co-localization of FtsK-RFP with the membrane (PCC > 0.7). Diad and tetrad cells were counted separately for the location of FtsK-RFP foci on the peripheral membrane (PM-D for diad and PM-T for tetrad), on the septal membrane (SM-D for diad and SM-T for tetrad), and on nucleoid (N-D for diad and N-T for tetrad). A significantly high foci density is seen on the septum as analyzed in both diads and tetrads (B). The *P*-values attained at 95% confidence intervals are depicted as (***) for <0.001. Cells grown in stationary conditions show FtsK-RFP expression in different locations- foci on the old or new septum, dispersed foci, and foci on both old and new septum making a cross pattern (scale bar-5μm) (C). The percentage of cells showing different cellular localization of FtsK-RFP foci in a population of ~200 cells was calculated and plotted (D). The images shown here are representative pictures of the experiments conducted at least three times.

The translational fusion of FtsK-RFP was expressed under a native promoter in D. radiodurans, and the cellular localization of drFtsK was observed microscopically. Interestingly, FtsK-RFP produced foci on the membrane (septal and peripheral) and the nucleoid (Fig. 6A). When we quantitated the number of FtsK-RFP foci in the cell, most of the foci were found to be on the septal membrane (SM) in comparison with the peripheral membrane (PM), and nucleoid (N), both in the diad and tetrad population (Fig. 6B). This result became more apparent when the cells’ growth rate was reduced by growing on agar plates and observing them under the microscope (Fig. 6C). Under these slow growth rate conditions, maximum FtsK-RFP foci density was seen on the septum. Around 200 cells were analyzed to determine the fraction of cells showing localization at the old or new septum, dispersed; and both on the new and old septum making a cross pattern (Fig. 6D). The formation of the new septum was determined based on the vancomycin staining, or the appearance of septum constriction as observed from the DIC pictures. Based on these observations, it can be speculated that FtsK protein localization is dynamic in D. radiodurans, showing on both nucleoid and septum depending on the growth stage of the cells.

### Time-lapse microscopy shows the movement of FtsK-RFP along the new septum in D. radiodurans.

**FIG 7:**
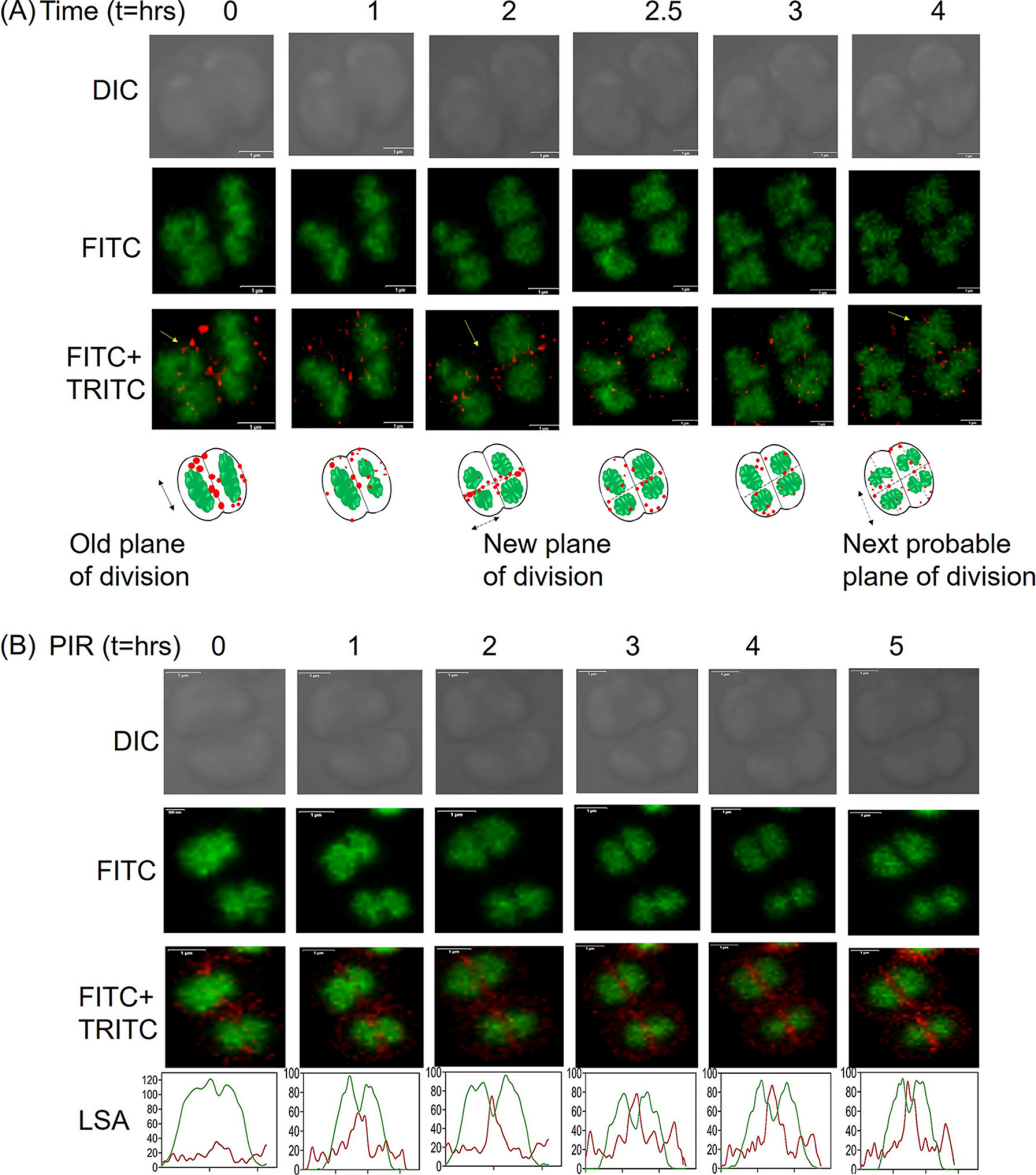
Cellular dynamics of deinococcal FtsK-RFP in normal and post-irradiation conditions. Time-lapse confocal microscopic images show FtsK-RFP (red foci) expressing cells stained with SYTO 9 green dye (green) during the normal condition (A) and during post-irradiation recovery (PIR) (B). The top panels indicate the cell in planar view at different time points (t = hr) in the DIC channel, the middle panels indicate the genome arrangement in the FITC channel and the bottom panels show the same cell with TRITC and FITC channel merged to see FtsK-RFP foci dynamics at different ‘t’ (scale bar-1μm). The dynamics of FtsK-RFP foci is schematically represented under normal condition. During PIR, line scan analysis (LSA) shows the increase in the intensity of FtsK-RFP along the emerging septum.

We observed that FtsK-RFP localization on the nucleoid and the septum is heterogenous, possibly because of the non-synchronous population of the cells. This prompted us to do time-lapse microscopy to observe the FtsK dynamics in dividing cells of D. radiodurans. Time-lapse microscopy was performed under both normal growth and the post-irradiation recovery phase (Fig. 7). Under normal conditions, at time point *t* = 0, maximum FtsK-RFP foci were aligned at the septum. With progression in the cell division, the alignment of the foci shifted to the newly forming septum perpendicular to the old septum (*t* = 2). This alignment was seen when the nucleoid separated into 2, and the septum constriction was just initiated. By *t* = 4, FtsK-RFP foci were seen to be moving to align along the next probable plane of division (Fig. 7A). In gamma radiation treated cells, the transition in the alignment of FtsK foci from the old septum to the new septum as the nucleoid divides was more apparent (Fig. 7B). At *t* = 0, FtsK-RFP foci were arranged along the membrane, mostly with prominently high foci density at the initiation sites of the new pole on septal and peripheral membranes. As time proceeded, most of FtsK-RFP foci positioned along the new division septum, perpendicular to the old one. Notably, line scan analysis (LSA) showed that the progression of FtsK alignment with the nucleoid separation process was very well synchronized (Fig. 7B). Thus, FtsK-RFP dynamically switched from old septa to new septa as the cell division progressed under both normal and irradiated conditions. Orientation of FtsK at the septum would be functionally essential to activate CDR so that the genome can be separated properly and segregated correctly into daughter cells. It was observed that the nucleoid divides into 2 daughter toroidal structures simultaneously with the FtsK dynamics from the old septum to the new septum. In E. coli, FtsK is known to bind to KOPS sequences on the genome and translocates toward the *dif* site. It also acts as a translocase by pumping out the septum-trapped DNA during chromosome separation and cell division (12). Our results obtained from the localization studies of drFtsK-RFP suggest a similar type of function in D. radiodurans also. Interestingly, there is an evident increase in the level of FtsK protein expression after irradiation (33). This could be explained by the transcriptome sequencing analysis where an increase in the *ftsK* transcript levels was seen after irradiation (33). D. radiodurans contains 6 to 8 copies of the genome which are damaged when treated with 6kGy gamma radiation. So, the participation of translocases like FtsK during the repair and resolution of the multipartite genome cannot be ruled out. There may be an increase in nuclear entanglement and chromosome dimers formation at the time of extensive synthesis and recombination of DNA during post-irradiation recovery. These cells may require the participation of FtsK and other proteins for activation of CDR. Thus, the results so far suggested that FtsK is dynamic in the cells under both normal and post-irradiation recovery conditions, and may be actively involved in the coordination of genome segregation and early cell division processes in this bacterium.

### Coordinated dynamics of FtsZ and FtsK in D. radiodurans.

**FIG 8:**
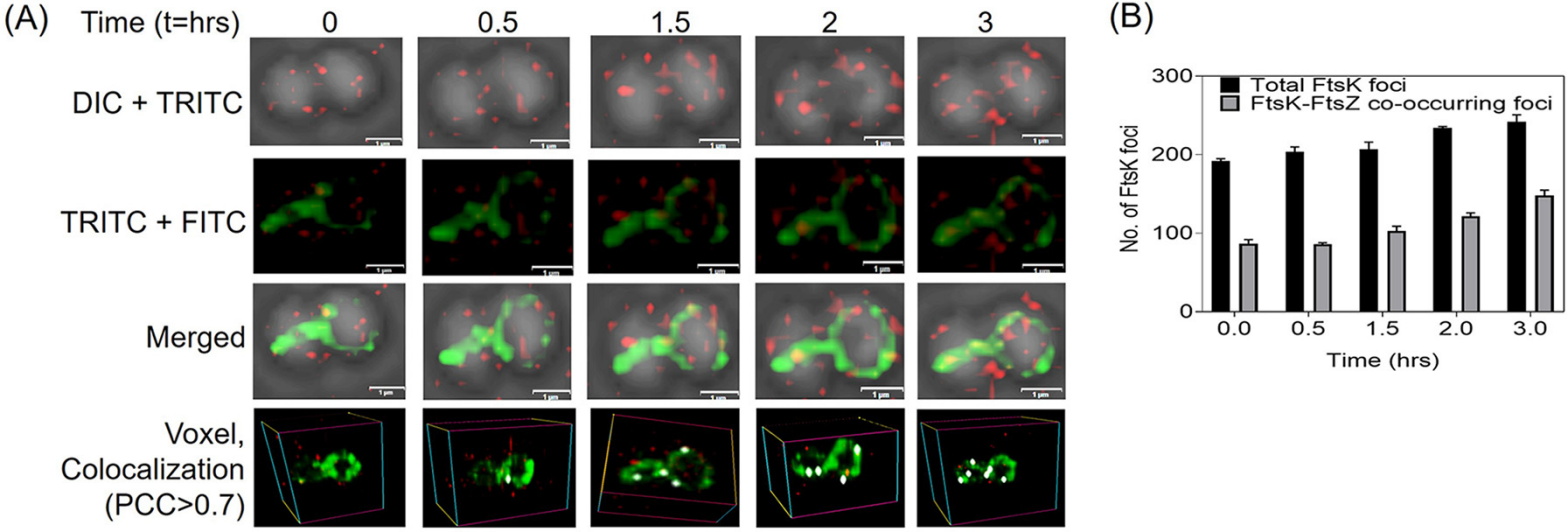
Co-ordinated cellular dynamics of cell division proteins FtsK and FtsZ in D. radiodurans. Time-lapse fluorescence microscopic images of cells expressing both FtsK-RFP and FtsZ-GFP show the dynamics of both the proteins in dividing cells under normal conditions. The top panel indicates the cell in planar view at different time points (t = hr) in the DIC+TRITC, the middle panels indicate the FITC+TRITC channel and merged images, and the bottom-most panel shows the same cell in voxel view with white foci showing the co-localization of FtsZ and FtsK (PCC > 0.7) (scale bar-1μm). The majority of FtsK-RFP (red) can be seen aligned at the division septa at *t* = 0.5 h and then moving to position themselves along the almost complete FtsZ ring at t = 3h (A). The total number of FtsK foci and FtsK-FtsZ co-occurring foci in a population of ~75 cells at time points of 0, 0.5 h, 1.5 h, 2 h, and 3 h were calculated and plotted (B).

The septum localization of FtsK-RFP and the presence of bent septum in the deletion mutant of *ftsK* led us to study the dynamics of FtsK with another cell division protein-FtsZ concurrently in D. radiodurans. For this, the cells expressing FtsK-RFP under the native promoter were expressed with FtsZ-GFP on the pVHZGFP plasmid. During time-lapse microscopy, ~75 cells were monitored for studying FtsZ and FtsK dynamics and analyzed. A single cell is shown in Fig. 8A. At the start of imaging (*t* = 0), the FtsZ ring was not complete, but as the cell growth proceeded, the FtsZ ring was formed in ~3 h (*t* = 3). The majority of FtsK-RFP could be seen aligning around the division septa at *t* = 0.5, and then moving to position itself along the growing FtsZ ring at *t* = 3 (Fig. 8B). This suggests that drFtsK localizes to the newly forming divisional septum after the FtsZ ring formation is completed. These microscopic results showed that FtsK aligns along the developing septum acting in coordination with FtsZ. Further, co-localization analysis of FtsZ and FtsK proteins suggest that both the proteins significantly co-localize in some places. Our microscopic results are the first reports showing coordinated dynamics of the important cell division proteins i.e., FtsZ and FtsK in D. radiodurans.

### FtsK interacts with divisome and segrosome components of D. radiodurans.

**FIG 9:**
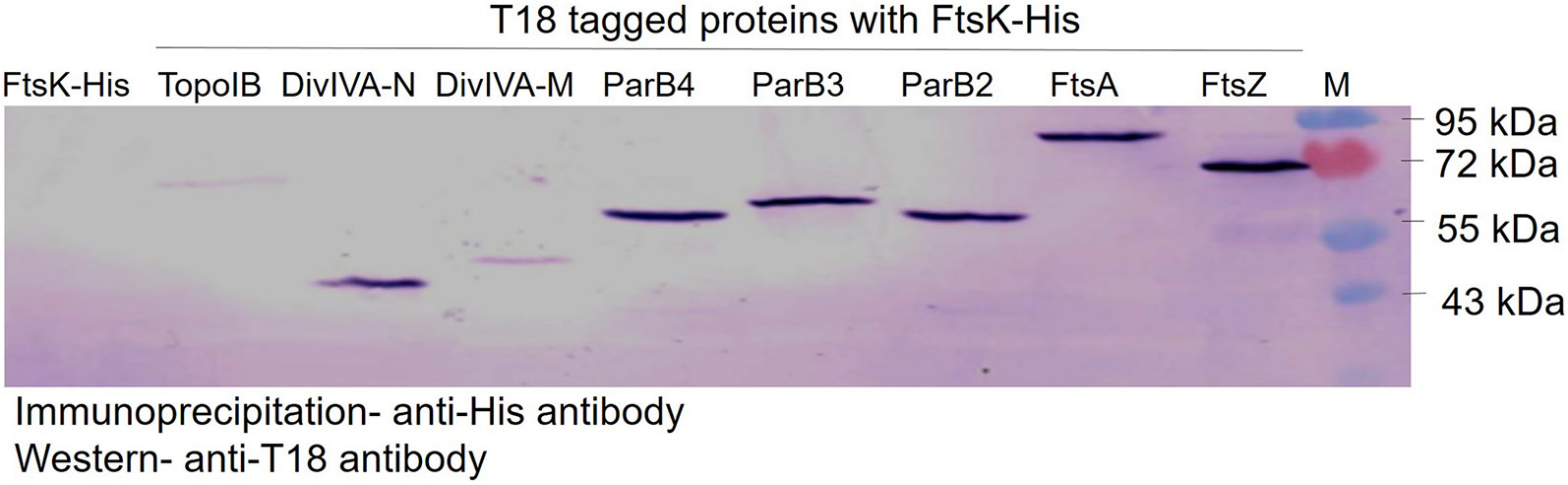
Interaction studies of deinococcal FtsK. Interaction of drFtsK and other proteins of D. radiodurans by co-immunoprecipitation assay followed by Western blot. His-tagged drFtsK and T18-tagged ParB2, ParB3, ParB4, TopoIB, FtsA, DivIVA, and FtsZ in E. coli strain BL21 were checked for protein-protein interactions by co-immunoprecipitation using antibodies against polyhistidine tag followed by immunoblotting using antibodies against T18 domain of CyaA. His-tagged FtsK expressing cells harboring only the pUT18 vector was taken as a negative control. The experiment was conducted three times and the representative picture was shown.

Results obtained so far suggest that FtsK is dynamic during different stages of bacterial growth, and plays an important role in both genome segregation and early cell division. The molecular basis of its dynamicity mainly relating to cell division and genome segregation would be worth understanding. Further, FtsK in other bacteria is known to interact with segrosome and divisome proteins (14). Therefore, the interaction of drFtsK with cell division and chromosome segregation proteins of D. radiodurans was checked by co-immunoprecipitation. It was observed that drFtsK interacts with deinococcal genome segregation proteins ParB2, ParB3, ParB4, and TopoIB; cell division proteins FtsZ, FtsA, and pole determining protein- DivIVA (Fig. 9). Its interaction with DivIVA and FtsZ could explain the reason behind the bent septum phenotype observed in *ftsK* mutants. Surprisingly, it does not interact with some of the deinococcal proteins like ParAs, GyrA, FtsW, and FtsE which are involved in nucleoid segregation and cell division. These results suggest that drFtsK, along with other genome segregation and cell division proteins, may be involved in macromolecular complex formation for its overall functions, and the possibility of this driving the dynamicity of FtsK independently of FtsZ cannot be ruled out in D. radiodurans.

## DISCUSSION

**FIG 10:**
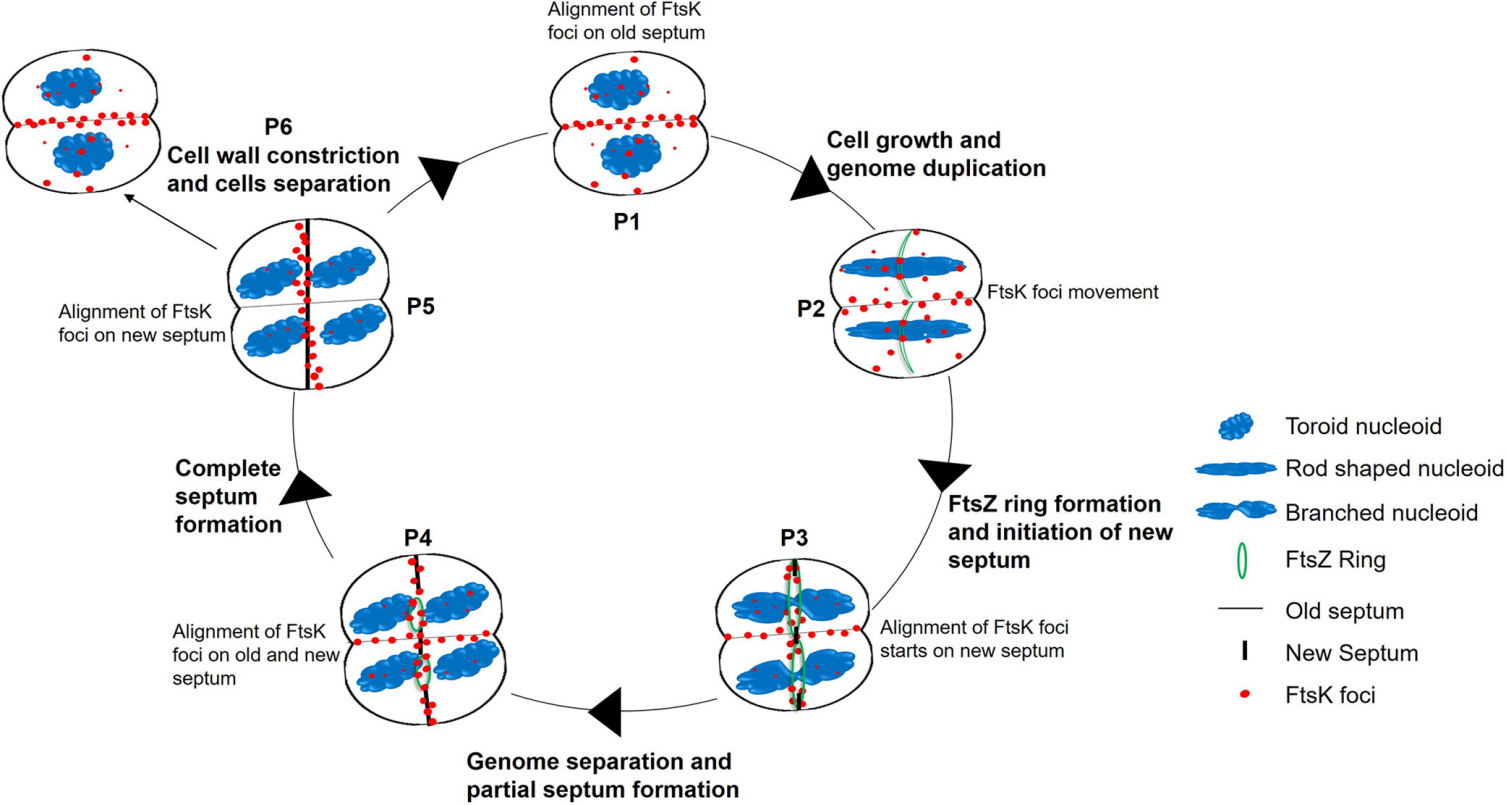
Schematic model of the dynamics of FtsK in D. radiodurans during cell division. Based on the biochemical assays and cellular localization studies, the probable role and dynamics of FtsK protein in exponentially growing D. radiodurans were proposed. FtsK forms foci on the nucleoid as well as on the membrane. The movement of FtsK can be linked with the different growth phases (P) as described. Initially, a maximum number of FtsK foci are aligned at the septum (P1). As the cell grows and the genome duplicates, FtsK foci move in the cell (P2). With the formation of the FtsZ ring and subsequent initiation of the new septa, the alignment of FtsK starts shifting perpendicularly from the old one to the new one (P3). When a new septum is partially formed, FtsK foci alignment is found on the old as well as new septum (P4). With complete new septum formation, FtsK moves entirely to the new septa before cell wall constriction starts (P5). The whole process repeats in the next cell division. The ATPase activity, sequence-specific DNA binding activity, and cellular interaction of FtsK with other segrosome and divisome proteins may aid in the smooth and coordinated progression of different cellular processes to maintain the genome stability in D. radiodurans.

D. radiodurans R1 is a multipartite genome harboring (MGH) bacterium having 2 chromosomes and 2 plasmids of 8 to 10 copies each per cell during the exponential growth phase (34, 35). The genome exists in a highly compacted nucleoid form which has been recently shown to dynamically change its shape during the cell division cycle (36). D. radiodurans exists in tetrads and grows by alternate planes of division where new cell division occurs in a plane perpendicular to the previous one. FtsK/SpoIIIE DNA translocases in bacteria are known to be involved in chromosome dimer resolution and pumping the trapped DNA through the newly formed septum during cell division or sporulation. Despite the critical role of FtsK, it is reported to be dispensable for division under certain conditions (37, 38). Most of these studies are done in single chromosome containing bacteria. Studies on the role of FtsK in MGH bacteria would be fascinating due to the complexities of multiple genome components present in the cell and among MGH, the work on FtsK have been reported only in V. cholerae (39, 40). Our genome analysis revealed that D. radiodurans encodes putative FtsK. Unlike V. cholerae, where both the chromosomes exist separately in the cells, the entire genetic material in D. radiodurans is packaged in the form of a doughnut-shaped toroidal nucleoid. Therefore, the possibility of FtsK functioning differently in maintaining the genome integrity of D. radiodurans cannot be ruled out. Here, we have brought forth some proof to indicate that drFtsK is active and is involved in both genome segregation and early cell division processes (Fig. 10). We demonstrated that recombinant FtsK is an ATPase with E. coli KOPS binding activity and could activate E. coli tyrosine recombinases XerCD *in vitro*, suggesting that drFtsK is functional in stimulating SSR. Fluorescence microscopy of cells expressing drFtsK-RFP showed the formation of multiple foci on the nucleoid as well as on the membrane. Multiple foci on the nucleoid could be attributed to the presence of KOPS on the genome, while foci formation on the membrane and/or septum could be attributed to FtsK transmembrane domain interacting with divisome components. A similar distribution of FtsK foci (~30 to 100 molecules of FtsK per cell) was reported in E. coli (41). Further, the localization pattern of FtsK in D. radiodurans seems to be cell cycle-specific where maximum protein intensity was observed on the septum in the dividing cells.

FtsK/SpoIIIE DNA translocases are multidomain and multifunctional proteins that are part of bacterial cell divisome machinery (42). In D. radiodurans, our earlier work showed the involvement of ParBs, TopoIB in genome segregation, and FtsZ, FtsA, and DivIVA in cell division (43–46). The co-immunoprecipitation results showed that drFtsK interacts with these proteins indicating its role in both of these processes. When different domain-specific drftsK deletion mutants were generated, the growth rate of mutants was significantly reduced during the early and exponential phases. Remarkably, the mutants delay growth and extend the growth phase to a region where the WT has already reached the stationary phase. Microscopy data showed improper membrane staining and membrane bulges in ftsK mutants. Membrane bulges were also observed earlier in S. aureus ΔftsK/ΔspoIIIE mutants (47). Recently, the roles of FtsK/SpoIIIE have been suggested in envelope remodeling in bacteria (48). All of this indicates the role of drFtsK in envelope reorganization during cell division and in mutants, where the lack of this function might have caused the delay in the growth rate (38). Previously, the movement of DNA through the tetrad compartments during PIR was shown to be necessary for the radiation resistance phenotype (27). The reduced growth rate was observed only during the initial time points (T1 period) in mutants exposed to gamma radiation, which could also be attributed to the delayed DNA translocation through the septum which may be required for accurate genome reassembly. Further, under normal growing conditions, the fast recovery of the mutants at the later phase can be speculated to be due to the bypass of FtsK, as seen earlier in E. coli by FtsA and FtsN interaction (49).

The genome segregation process in E. coli progresses from the *ori*-to-*ter* loci, and FtsK protein was actively involved in the “ter” locus positioning at the mid-cell region (50). In V. cholerae with 2 divergent *difs*, chromosome resolution sites were identified for 2 chromosomes, and both are activated by FtsK (39). In D. radiodurans also, genome sequence analysis has revealed putative “*dif”* sites and 6 putative tyrosine recombinases on both primary and secondary chromosomes, which show around 25 to 30% similarity to E. coli tyrosine recombinases (see the data at https://barc.gov.in/publications/mbio/dna_mp/mBio01742-22R1.pdf). An independent study will be conducted to check the functional interacting partners of drFtsK among these recombinases and their role in chromosome dimer resolution at “*dif”* sites.

Recent electron cryo-microscopy (cryo-EM) structure studies of dsDNA bound FtsK_αβ_ from P. aeruginosa revealed changes in different conformational states of ATPase domains in homo-hexameric rings that generate the translocation movement on DNA (51). drFtsK also forms homo-hexamers and the time-lapse microscopy results showed that FtsK-RFP is dynamic, orientating its translocating activity along the new septum from the old septum in dividing cells. Based on the observed results, FtsK movement can be linked to the different growth phases (P), as described earlier in exponentially growing D. radiodurans (36), where the active role of FtsK protein in the smooth coordination of genome segregation and cell division processes can be thought of (Fig. 10). As shown in the pictorial form, during the exponential growth phase, when a cell is divided into 2 forming the diad, most of the FtsK aligns on the old septum (P1) (Fig. 10). During the subsequent cell growth and genome duplication and elongation, FtsK disperses on the genome (P2) (Fig. 10). At the early cell division stage, when FtsZ ring formation and initial constriction occur, FtsK alignment shifts from the old to the new septum (P3) (Fig. 10). Foci alignment on the new septum is more prominent at the later stages of genome segregation and cell septum formation (P4 and P5) (Fig. 10). Thus, FtsK may be involved in marking the new septum, and each cell divides into 2, forming a tetrad after complete septum formation and cytokinesis (P6) (Fig. 10). All the results suggest that the dynamic multiprotein interactions coordinate accurate segregation of duplicated intertwined circular genome elements, as well as the next plane of cell division in this coccus. In conclusion, the drFtsK characterized in this report is a multifunctional protein having ATPase and DNA binding activities, and has a role in the determination of cell architecture, in general, and a faithful inheritance of its multipartite genome system.

## MATERIALS AND METHODS

### Bacterial strains, growth conditions, plasmids, and materials.

All bacterial strains and plasmids used in this study are listed at https://barc.gov.in/publications/mbio/dna_mp/mBio01742-22R1.pdf. D. radiodurans R1 (ATCC13939) was a kind gift from Professor J. Ortner, Germany (52). This bacterium was grown in Tryptone (0.5%), Yeast extract (0.3%) and Glucose (0.1%) (TYG) medium at 32°C. E. coli strains were grown in LB broth (1% tryptone, 0.5% yeast extract, and 1% sodium chloride) at 37°C. E. coli strain DH5α was used for cloning purposes and maintenance of all the plasmids. For the expression of recombinant proteins, E. coli strain BL21(DE3) pLysS was used. Cells harboring pET28a (+) derivatives or Bacterial Two-Hybrid System (BACTH) vectors and their derivatives were maintained in the presence of the respective antibiotic selection pressure. Shuttle expression vector pVHSM (53) and its derivatives were maintained in the presence of spectinomycin in E. coli and D. radiodurans cells (70 μg/mL and 40 μg/mL, respectively). Deinococcal cells harboring suicide vector- pNOKOUT (54) derivatives were grown in the presence of kanamycin (8 μg/mL). All the enzymes and molecular biology grade chemicals were purchased from Sigma Chemical Company, and Merck India Pvt. Ltd. Isopropyl-β-d-1-thiogalactopyranoside (IPTG), DAPI, Nile red, and vancomycin hydrochloride (unlabeled) were purchased from Merck Inc. Vancomycin BODIPY FL conjugate (labeled) was purchased from Invitrogen. SYTO 9 green fluorescent nucleic acid stain was purchased from Sigma-Aldrich. Antibody against T18 (SC-33620) domain of CyaA of Bordetella pertussis, was obtained commercially from Santa Cruz Biotechnology, Inc. Antibody against polyhistidine tag was procured from Sigma. Radiolabelled nucleotides were acquired from the Board of Radiation and Isotope Technology, Department of Atomic Energy (DAE), India (BRIT, India).

### Bioinformatic analysis and molecular modeling.

The D. radiodurans genome encodes a putative FtsK (locus tag E5E91_ RS02025, old locus tags- Dr_0400 and Dr_0401) on chromosome I, annotated as DNA translocase FtsK (hereafter called drFtsK). The full-length FtsK sequence of D. radiodurans and its other characterized homologs in B. subtilis (SpoIIIE_Bs), E. coli (FtsK_Ec), P. aeruginosa (FtsK_Pa), S. aureus (FtsK_Sa), and V. cholerae (FtsK_Vc) were retrieved from the NCBI genome database. Multiple sequence alignment and secondary structure prediction were performed with the Promals3D online server (55). The neighbor-joining phylogenetic tree (without distance corrections) between deinococcal FtsK and known FtsK family proteins was constructed using the PHYLIP program. After alignment, the Walker box (walker A ‘P-loop’ and walker B) ATP binding motif and DNA binding motif were searched. Structural models of drFtsK protein domains (motor domain and gamma domain) were constructed by the I-TASSER server (http://zhanglab.ccmb.med.umich.edu/I-TASSER/). The models were validated by the Swiss model workspace. Templates used for the modeling of drFtsK domain structures were derived from the known structures of respective E. coli FtsK motor domain (PDB ID: 2ius) and gamma domain (PDB ID: 2j5p). The modeled structures of the deinococcal FtsK domains were superimposed with E. coli FtsK domain structures (2ius and 2j5p) using PyMOL software and RMSD, and TM score were analyzed which indicated high similarity between the models. KOPS motifs were searched across the D. radiodurans genome by DistAMo online tool, shown at https://barc.gov.in/publications/mbio/dna_mp/mBio01742-22R1.pdf (56).

### Cloning, expression, and purification of recombinant proteins.

A list of all the primers used for constructing recombinant plasmids and generating deletion/insertion mutants are mentioned at https://barc.gov.in/publications/mbio/dna_mp/mBio01742-22R1.pdf. The open reading frames (ORF) of drFtsK (Dr_0400/0401), drFtsKΔN (Dr_0400), drFtsKγ, ecFtsKγ, ecXerC, and ecXerD were PCR amplified from D. radiodurans or E. coli genome (as applicable) using sequence-specific primers. PCR products were purified and ligated at NdeI and BamHI sites in pET28a (+) to produce the respective plasmids (see the data at https://barc.gov.in/publications/mbio/dna_mp/mBio01742-22R1.pdf). These plasmids were transformed into E. coli BL21(DE3) pLysS. Induction of expression and preparation of cell extract was performed as reported earlier (57). Purification of proteins was done by nickel affinity chromatography. For that, the cell extract was loaded onto NiCl_2_ charged-fast-flow-chelating-Sepharose column (GE Healthcare) which was pre-equilibrated with buffer (20 mM Tris-HCl, 300 mM NaCl, 10% glycerol) containing 10 mM imidazole. Extensive washing of column was done with 20 volumes of buffer A having 50 mM imidazole. Stepwise elution of recombinant proteins was done using 100 mM, 200 mM, and 250 mM imidazole in buffer A, and analysis of each fraction was carried out on 10% SDS-PAGE. Fractions containing the desired proteins at the expected size were pooled and dialyzed in buffer A containing 100 mM NaCl, and proceeded for HiTrap Heparin HP column purification. drFtsKΔN protein quality was examined by Circular dichroism (CD) spectroscopy in buffer (20 mM Tris-HCl, pH 7.6, 100 mM NaCl) using JASCO, J815 with ~0.2 mg/mL, as described earlier (43). The oligomeric status of the protein (5 μM) was checked by Dynamic Light Scattering (DLS) in the same buffer as mentioned before, using the Malvern Panalytical Zetasizer nano range instrument with/without KOPS DNA (100 nM) and ATP (0.5 mM).

### ATPase activity assay.

ATPase activity detection was done by thin-layer chromatography method to track the release of [^32^P]-αADPs from [^32^P]-αATPs and by malachite green assay to quantify the amount of inorganic phosphate (Pi) released as described in (43). Briefly, different concentrations of drFtsKΔN were added with 30 nM [^32^P] -α ATP in a buffer containing 20 mM Tris (pH 7.6), 75 mM KCl, 2 mM MgCl_2_, and incubated at 37°C for 0.5 h. The reaction was discontinued using 10 mM EDTA, and 1 μL of each reaction mixture was spotted on the PEI-Cellulose F+ TLC sheet. Components were separated on solid support after being air-dried in a buffer containing 0.75M KH2PO4/H3PO4 (pH 3.5), and an autoradiogram was developed. For the colorimetric quantitative assay, ATPase/GTPase activity assay kit (Sigma-Aldrich) was used with/without KOPS DNA (100 nM), and μmole Pi was released per min per μL was calculated. Data were plotted using the GraphPad Prism 6 software.

### Protein-DNA interaction study.

The DNA binding activity of drFtsKΔN was checked by electrophoretic mobility shift assay (EMSA) as described in (58). In brief, ssDNA containing E. coli *dif* and KOPS (72mer) or E. coli *dif* (40mer) sequence were radiolabeled with [^32^P]-γATP using T4 polynucleotide kinase (31). Forward strands were annealed with the complementary strands to yield radiolabeled dsDNA (*dif* and *dif*+KOPS). Approximately 30 nM radiolabeled dsDNA was incubated with different concentrations of drFtsKΔN (0 to 2 μM) in a buffer containing 50 mM Tris-Cl (pH 8.0), 75 mM NaCl, 5 mM MgSO4, and 0.1 mM DTT for 30 min at 37°C with/without 2 mM ATP. The reaction mixture was loaded on 8% native PAGE gel, the gel was dried, and the autoradiogram was developed.

### Recombination assay.

An *in vitro* recombination reaction was performed as reported by (31). A total of 1 nM radiolabeled *dif* fragment and 10 nM dif- KOPS fragment was incubated with 150 nM ecXerC, 30 nM ecXerD in buffer containing 10 mM MgCl_2,_ 20 mM Tris/HCl pH 7.6. To this, 40 nM FtsK variants (drFtsKΔN, drFtsKγ, and ecFtsKγ) were added separately and incubated for 2 min. The reaction was monitored in the presence/absence of 2 mM ATP for 30 min at 37°C. The reaction was stopped by adding 0.1% SDS and 0.1 mg/mL proteinase K, and the mixture was run through a 7% denaturing PAGE gel containing 0.1% SDS in 1X TBE buffer. The gel was dried, and an autoradiogram was developed. Band intensities on the autoradiogram were estimated densitometrically by ImageJ 2.0 software and % recombination product was calculated.

### Generation of drftsK deletion mutants and ftsK-rfp knock-in mutant.

For creating deletion mutants of the different domains of FtsK in D. radiodurans, the respective regions of the coding sequence of *ftsK* (see the data at https://barc.gov.in/publications/mbio/dna_mp/mBio01742-22R1.pdf) were replaced by *nptII* as described earlier (54). In brief, ~1 kb upstream and ~1 kb downstream sequences to the target region were PCR amplified from D. radiodurans genomic DNA using sequence-specific primers (as given at https://barc.gov.in/publications/mbio/dna_mp/mBio01742-22R1.pdf). The upstream fragment was cloned at ApaI and EcoRI sites while the downstream fragment was cloned at BamHI and XbaI sites into a suicide pNOKOUT vector (54) to produce pNKFKUD, pNKFK_MC_UD, and pNKFK_N_UD plasmids (see the data at https://barc.gov.in/publications/mbio/dna_mp/mBio01742-22R1.pdf). The recombinant plasmids were linearized with *Xmn*I and transformed into D. radiodurans separately. Transformants obtained were plated on TGY plates containing kanamycin (8 μg/mL) and grown for many generations under necessary antibiotic pressure to obtain homozygous insertion of *nptII* and replacement of the target portions of *ftsK* in the whole deinococcal genome. This was confirmed by PCR amplification using *ftsK* domain-specific primers as well as antibiotic (*npt*II) cassette-specific primers (see the data at https://barc.gov.in/publications/mbio/dna_mp/mBio01742-22R1.pdf). The homozygous replacement of the target genes with the *nptII* cassette was accomplished, and these cells with genotype *ftsK*::*nptII*, *ftsKN*::*nptII*, *ftsKMC*::*nptII* were denoted as Δ*ftsK*, Δ*ftsKN*, Δ*ftsKMC* respectively. The *ftsKN*::*nptII* mutant has a deletion of N-terminal 1 to 163 amino acids of drFtsK (M1-L163), *ftsKMC*::*nptII* mutant has a deletion of 295 to 1209 amino acids of drFtsK (D295-K1209), and *ftsK*::*nptII* has a deletion of full-length drFtsK (M1-K1209).

For expression of *ftsK-rfp* under the native promoter of *ftsK*, the replacement of chromosomal copy of *ftsK* with *ftsK-rfp* was carried out using a similar approach as mentioned above. The translational fusion of FtsKγ-RFP was generated by cloning the *ftsK* gamma domain (*ftsK*γ) in pDsRed vector to yield pDsRedFKγ. Further, *ftsKγ-rfp* (from pDsRedFKγ) was cloned upstream to *nptII* and downstream *ftsk* sequence was cloned downstream to *nptII* in pNOKOUT to yield pNKFKγRD. This was transformed into D. radiodurans to obtain cells with genotype *ftsK::ftsK-rfp*, which expressed FtsK-RFP under the native promoter.

### Growth studies of different deletion mutants of ftsK.

D. radiodurans R1 WT and *ftsK* mutants were exposed to 6kGy gamma radiation as described in (59). Briefly, overnight grown bacterial cultures (with/without kanamycin; 8 μg/mL) were washed and suspended in sterile phosphate-buffered saline (PBS). These cells were then exposed to 6 kGy gamma radiation at a dose rate of 1.5 kGy/h (Gamma Cell 5000, ^60^Co, Board of Radiation, and Isotopes Technology). Equal numbers of IRR and control UI cells were grown in TYG medium (with/without kanamycin; 8 μg/mL) in 96-well microtiter plates after washing with PBS (Nunclon; Sigma-Aldrich). Optical density at 600 nm was measured to examine the growth in replicates at 32°C for 18 h using Synergy H1 Hybrid multi-mode microplate reader. The growth curves were fitted using the spline regression model. Statistical analysis was carried out using the statistical programs R, http://www.r-project.org/, the “spline” package was used to generate the spline regression-based models in R. The model was developed using linear b-splines with 2 knots producing 3 intervals (1,2,3). Fixed time intervals were chosen as 0 to 5 h (T1), 5 to 10 h (T2), and 10 to 18 h (T3). The linear growth rates were computed by the change in absorbance (OD) per interval. UI samples were compared with R1-UI as control, and IR samples were compared with R1-IRR as control. The generation time of wildtype and *ftsk* mutants were calculated as mentioned in (60). Graphs of growth rate coefficient v/s strain at each time interval and generation time were plotted using GraphPad Prism 6.0. Significance value (*P* value) obtained at 95% confidence intervals were considered to be significantly different.

### Microscopy studies of different deletion mutants of ftsK.

Confocal microscopy was done on IX3SVR using an Olympus IX83 inverted microscope with the laser beams focused on the back focal plane of a 100 × 1.40 NA oil-immersion apochromatic objective lens (Olympus) as described earlier (44). The time sequence and intensity of laser illumination at samples were adjusted using the installed FLUOVIEW software. For imaging, a series of Z-planes were acquired at every 400 nm using a motorized stage, and then z-stacking was done to create 3D images. In brief, D. radiodurans R1 WT cells and different deletion mutants of *ftsK* in exponential phase at 3 time points, 6 h, 9 h, and 14 h were taken (Fig. 5 and data at https://barc.gov.in/publications/mbio/dna_mp/mBio01742-22R1.pdf). The cells were washed twice with phosphate-buffered saline (pH 7.4). These cells were stained with Nile red (1 mg/mL) for membrane and DAPI (40,6-diamidino-2-phenylindole, dihydrochloride) (0.2 mg/mL) for genome for 10 min on ice and then washed thrice with PBS. Cells were then mounted on a 1% agarose bed on glass slides and imaged. Fluorescence from Nile Red/RFP tagged protein was detected using tetramethylrhodamine isothiocyanate (TRITC) filter with 542/562 nm, excitation/emission wavelength. Fluorescence from DAPI was detected using its filter with 402/460 nm, excitation/emission wavelength. Each image is represented in separated, as well as merged channels. The brightness and contrast of all images were tuned using Adobe Photoshop 7.0. For quantification of image patterns, 200 to 300 cells from two to three isolated microscopic fields were taken in independent experiments and analyzed for necessary attributes. Image analysis and other cell parameters were determined using automated Olympus CellSens software. Data obtained was plotted using GraphPad Prism 6.0.

### Localization of FtsK and FtsZ in WT and ΔftsK mutant.

For studying the localization of FtsK-RFP foci, unlabeled vancomycin and labeled (Van-FL-BIODPY) were combined in the ratio of 1:1 (0.5 μg/mL) to stain the membrane as mentioned before (61), and exponential phase cells expressing FtsK-RFP under native promoter were grown for 90 to 120 min. These cells were washed twice with phosphate-buffered saline (pH 7.4) and stained with DAPI. Fluorescence from Van-FL-BODIPY/GFP tagged protein was detected using a FITC filter with 475/530, excitation/emission wavelength. Image analysis was done as described above. Around 150 diad and 150 tetrad cells were counted separately for the quantification of localization of FtsK-RFP foci on the nucleoid and the peripheral membrane or the septal membrane. Data were plotted as a scatterplot in GraphPad Prism 6 software. For FtsZ localization studies, the recombinant plasmid pVHZGFP expressing FtsZ-GFP was transformed in WT and Δ*ftsK*-mutant cells of D. radiodurans. Cells were grown overnight, and cells equivalent to 0.05 to0.1 OD_600_ were diluted with a fresh medium containing the required antibiotic and induced with 10 mM IPTG overnight.

### Growth phase-dependent studies on FtsK and FtsZ dynamics.

Time-lapse imaging was done for the monitoring of the dynamics of FtsK with respect to genome movement, cell growth, and division. Cells expressing FtsK-RFP were stained with 150 nM SYTO 9 dye and PBS washed cells were placed on an agarose pad made in 2X-TYG and constructed with air holes for oxygenation of cells. Fluorescence emission was grabbed with DM-488/561 dichroic mirror and corresponding single-band emission filters at different time intervals. Images were taken for a period of 4 h at intervals of 1 h using very low laser power (561 nm and 488 nm). For monitoring FtsK localization and dynamics under gamma radiation exposure, the cells were treated with 6kGy radiation and grown for 1 h with constant shaking. Then, the cells were visualized as mentioned above. We did a line scan analysis (LSA) for each time point through CellSens software. In LSA, we scanned the fluorescence intensity of SYTO 9 and FtsK-RFP signals across the growing/developing septum to find the relative intensity of the genome and FtsK-RFP. For studying FtsK and FtsZ dynamics, cells expressing FtsK-RFP under native promoter were transformed with pVHZGFP and similarly proceeded for time-lapse microscopy as explained above. All the co-localization studies were done using built-in CellSens Software and the represented co-localization patterns showed Pearson’s correlation coefficient (R[r]) > 0.7-1.0 and Overlap coefficient (R) of 0.9 to 1.0. Co-localization was represented as white foci in the images. A total of 76 cells were analyzed at each time point to determine the number of FtsK foci co-occurring with FtsZ. Values obtained were plotted using GraphPad prism-6.0.

### Protein-protein interaction study.

The possible interaction of drFtsK with other deinococcal proteins of cell division like FtsZ, FtsA, and DivIVA, and genome segregation proteins like TopoIB, ParB2, ParB3, and ParB4 was monitored in surrogate E. coli by using the co-immunoprecipitation method. The recombinant pUT18 plasmids that were already used in the earlier studies (45, 46, 62, 63) were transformed in E. coli BL21(DE3) pLysS strain expressing histidine-tagged FtsK (pETFtsK). E. coli cells co-expressing histidine-tagged FtsK with T18-tagged protein in separate combinations were obtained at the log phase and cell extracts were prepared. Total proteins were immunoprecipitated using anti-polyhistidine antibodies and the possibility of T18 tagged partners’ presence in immunoprecipitate was checked using monoclonal antibodies against the T18 domain of CyaA as depicted earlier (62, 63). Hybridization signals were detected with anti-mouse secondary antibody labeled with alkaline phosphatase using NBT/BCIP substrates (Roche Biochemical, Mannheim).

### Statistical analysis.

All the statistical analysis was done using Student's *t* test. Significance value (*P* value) obtained at 95% confidence intervals are depicted as **** for *P* value <0.0001, *** for *P* value <0.001, ** for *P* value of 0.05-0.001, and * for *P* value <0.05.
